# A Five-Ingredient Nutritional Supplement and Home-Based Resistance Exercise Improve Lean Mass and Strength in Free-Living Elderly

**DOI:** 10.3390/nu12082391

**Published:** 2020-08-10

**Authors:** Mats I. Nilsson, Andrew Mikhail, Lucy Lan, Alessia Di Carlo, Bethanie Hamilton, Kristin Barnard, Bart P. Hettinga, Erin Hatcher, Milla G. Tarnopolsky, Joshua P. Nederveen, Adam L. Bujak, Linda May, Mark A. Tarnopolsky

**Affiliations:** 1 Department of Pediatrics, McMaster University Medical Center, Hamilton, ON L8N 3Z5, Canada; mnilsson7714@gmail.com (M.I.N.); mikhaiai@mcmaster.ca (A.M.); lucy.lan2012@gmail.com (L.L.); dicarlae@mcmaster.ca (A.D.C.); hamiltob@mcmaster.ca (B.H.); barnardk@hhsc.ca (K.B.); hatchere@hhsc.ca (E.H.); millaskier@gmail.com (M.G.T.); nedervj@mcmaster.ca (J.P.N.); maylind@mcmaster.ca (L.M.); 2 Exerkine Corporation, McMaster University Medical Center, Hamilton, ON L8N 3Z5, Canada; bart.hettinga@exerkine.com (B.P.H.); adambujak01@gmail.com (A.L.B.); 3 Department of Kinesiology, McMaster University, Hamilton, ON L8N 3Z5, Canada

**Keywords:** sarcopenia, resistance exercise, multi-ingredient supplement, whey, creatine, omega-3, vitamin D, randomized clinical trial, COVID-19

## Abstract

Old age is associated with lower physical activity levels, suboptimal protein intake, and desensitization to anabolic stimuli, predisposing for age-related muscle loss (sarcopenia). Although resistance exercise (RE) and protein supplementation partially protect against sarcopenia under controlled conditions, the efficacy of home-based, unsupervised RE (HBRE) and multi-ingredient supplementation (MIS) is largely unknown. In this randomized, placebo-controlled and double-blind trial, we examined the effects of HBRE/MIS on muscle mass, strength, and function in free-living, older men. Thirty-two sedentary men underwent twelve weeks of home-based resistance band training (3 d/week), in combination with daily intake of a novel five-nutrient supplement (‘Muscle5’; M5, *n* = 16, 77.4 ± 2.8 y) containing whey, micellar casein, creatine, vitamin D, and omega-3 fatty acids, or an isocaloric/isonitrogenous placebo (PLA; *n* = 16, 74.4 ± 1.3 y), containing collagen and sunflower oil. Appendicular and total lean mass (ASM; +3%, TLM; +2%), lean mass to fat ratios (ASM/% body fat; +6%, TLM/% body fat; +5%), maximal strength (grip; +8%, leg press; +17%), and function (5-Times Sit-to-Stand time; −9%) were significantly improved in the M5 group following HBRE/MIS therapy (pre vs. post tests; *p* < 0.05). Fast-twitch muscle fiber cross-sectional areas of the quadriceps muscle were also significantly increased in the M5 group post intervention (Type IIa; +30.9%, Type IIx, +28.5%, *p* < 0.05). Sub-group analysis indicated even greater gains in total lean mass in sarcopenic individuals following HBRE/MIS therapy (TLM; +1.65 kg/+3.4%, *p* < 0.05). We conclude that the Muscle5 supplement is a safe, well-tolerated, and effective complement to low-intensity, home-based resistance exercise and improves lean mass, strength, and overall muscle quality in old age.

## 1. Introduction

Biological aging is inevitable and intrinsic and underlies the progressive cell deterioration that begins after the reproductive peak in humans [1]. Lifestyle-associated factors, such as physical activity (PA) and dietary patterns, are considered extrinsic and are therefore modifiable, but are also negatively impacted by the aging process. Collectively, these intrinsic and extrinsic factors govern the rate of deterioration of the critical organ and cell systems that regulate oxygen supply, energy production, contractile activity, and movement.

Age-related loss of skeletal muscle (SM) mass, strength, and function is known as sarcopenia [2,3] and is associated with an increased fall risk [4,5], disability [6,7], institutionalization [7], and all-cause mortality [7,8,9]. Pioneering studies by Lexell et al. showed that SM cross-sectional area (CSA), fiber size, and fiber number are reduced by 20–40% from adulthood to old age [10,11], paralleling the loss of aerobic fitness (VO_2max_), glucose tolerance, and anabolic hormones [12,13,14]. A conservative estimate is that >50 million of individuals are affected by sarcopenia and that this number will rise to >200 million in the next 40 years [15], with major economic implications for global health-care systems [16].

Considering the growing proportion of elderly and the concurrent rise in age-associated chronic disease, research efforts are warranted to develop innovative, cost-saving, and effective countermeasures. Over the last 60 years, it has been shown that regular PA decelerates the intrinsic aging process of most, if not all, organ systems, reducing all-cause mortality and extending lifespan by 3–10% [1]. Although much focus has been placed on the cardiovascular system, the significance of SM preservation is demonstrated by the fact that strength and power predict all-cause mortality independent of aerobic capacity [17,18]. It is therefore well-accepted that maintenance of the musculoskeletal system is vital for physical functioning, independence, and quality of life in old age [19,20,21,22,23].

Resistance exercise (RE) is the main, natural anabolic stimulus for SM growth, with 2–3 d/week at moderate-to-vigorous intensity sufficient to reduce mortality risks and musculoskeletal dysfunction even in the “oldest old” [24,25,26,27]. Petersen et al. conducted a meta-analysis of 49 studies with 1328 mixed gender participants aged 50–83 years old and concluded that ~20 weeks of RE (range 10–52 weeks) at ~75% 1 RM (range 50–80%) augments lean mass with ~1.1 kg on average [28]. Another review concluded that RE of the same duration and intensity increases muscle strength and type I/type II fiber areas by ~70% and ~20%, respectively [29]. Older adults typically gain ~5.5% in muscle strength for each incremental increase in RE intensity over 60% one-repetition maximum (1-RM) [30], and improvements in muscular endurance and power may be even greater than muscle strength [31,32].

While supervised RE generally yields superior results compared to the unsupervised counterpart [33,34], both types effectively mitigate age-related SM dysfunction [34,35,36]. However, long-term benefits of traditional RE forms are usually limited by low adherence and discontinuation of training efforts [37]. Non-traditional alternatives, such as home-based, low-impact RE, may circumvent the typical barriers to exercise participation, including fear of injury, pain, financial constraints, and/or lack of time, and therefore increase long-term adherence. Indeed, home-based RE alternatives have never been more applicable than during the COVID-19 (SARS-CoV-2) pandemic with general access to parks, sporting facilities, and gymnasiums being limited due to restrictions on physical contact and social gatherings. People who are at high risk for severe complications due to SARS-CoV-2, for example elderly and polymorbid individuals, are already predisposed to disuse syndrome and particularly vulnerable to these restrictions [38], with the forced lockdown apparently reducing PA levels significantly among those ≥55 years of age [39].

Despite the profound benefits of RE across all age-groups, there is some evidence of a blunted anabolic response to contractile activity with aging (e.g., anabolic resistance) [40,41,42,43,44]. Consequently, a combination of RE with other interventional strategies may be necessary to maintain muscle health in old age. For example, the synergism between branched-chain amino acids (BCAAs) and contractile activity is well-established [45], with leucine being the most potent of all BCAAs in activating anabolic pathways, although the majority of essential amino acids (EAAs) are necessary for muscle protein synthesis (MPS) [46,47,48]. Indeed, the provision of EAAs (≥10.9 g) and leucine (≥2.7 g) potentiates the anabolic response to RE, and various animal protein sources, such as whole milk (≥31 g), whey (≥25 g), and caseinate (≥30 g), contain sufficient anabolic factors to induce SM gains in combination with RE [49,50,51]. Plant-based options (for example soy) generally require higher dosages and have lower anabolic potential compared to animal-based counterparts [51,52]. Collectively, the literature suggests that a higher single dose (≥40 g whey), multiple daily dosing, increased total protein intake (1.2–1.6 g protein/day), or provision of additional anabolic nutrients are necessary to maximize MPS and maintain SM in old age [40,41,53,54,55,56,57,58].

The idea of using multi-ingredient supplementation (MIS) mainly rests on the simultaneous targeting of several metabolic and signaling pathways to potentiate SM gains, including the major growth regulatory processes within the muscle cell (e.g., protein synthesis, protein degradation, and satellite cells), energy production, contractile function, and/or recovery. As MIS stimulates several processes simultaneously, it theoretically ‘circumvents’ the main limitation of using traditional, single-nutrient strategies to maintain SM in old age, e.g., anabolic resistance. A myriad of supplements are purported to provide significant benefits for SM strength, performance, and recovery, but relatively few are supported by sound evidence from randomized clinical trials (RCTs). Ultimately, there is a need for RCTs to assess the safety and efficacy of a vast majority of supplements due to the lax regulatory framework generally surrounding these products, increasing the risk of false advertising, unsubstantiated claims, and health side-effects [59]. Specifically, the diverse nutritional and pharmacological approaches to attenuate sarcopenia reflect the complex etiology of the condition [60], but only a handful of these are safe, well-tolerated, and could be prescribed for long-term benefits.

Beyond protein supplementation, vitamin D [61,62], polyunsaturated fatty acids (n-3 PUFAs; EPA and DHA [63,64]), and creatine [65,66,67,68] have documented SM benefits and may be safely combined with exercise therapy in older adults. The efficacy of protein-based MIS in older adults was recently demonstrated by Bell et al., who found that whey protein, creatine, EPA/DHA, and vitamin D enhanced lean mass, strength, cognition, n-3 index, and lowered markers of inflammation in older males [53,69,70]. Specifically, the participants underwent six weeks of MIS alone (Phase 1), followed by 12 weeks of MIS combined with supervised, whole-body RE (2 d/week, 80% 1-RM) and HIIT (1 d/week) (Phase 2), and MIS was superior to placebo in both phases. Importantly, there were no reported side-effects associated with MIS, such as kidney or gastrointestinal issues, despite using relatively high dosages of total daily protein (60 g) and creatine (5 g). Two recent meta-analyses by O’Bryan et al. (1397 participants) and Naclerio et al. (192 participants), respectively, have also confirmed that protein-based MIS is safe and augments both fat-free mass and strength in resistance-trained, older adults [55,71]. Lastly, the notion that protein-based MIS may not add benefits over protein supplementation alone may largely be of academic interest considering that commonly used nutrients in MIS, such as EPA/DHA, vitamin D, and calcium, have documented benefits that extend beyond SM maintenance [69,70]. For example, with relevance to the current COVID-19 pandemic, it has become increasingly clear that malnutrition in general, and specifically vitamin D insufficiency, is associated with worse outcomes and higher mortality rates in COVID-19 patients [72].

In this randomized, placebo-controlled RCT, we wanted to test the utility of a five-ingredient protein-based MIS (‘Muscle5’; M5) in combination with low-intensity, home-based RE (HBRE; whole-body elastic band training) for maintenance of SM mass, strength, performance and muscle quality (MQ) in sedentary, free-living elderly. Specifically, our aim was to test the efficacy of HBRE (3 d/week) when combined with a multi-nutrient supplement containing lower amounts of total protein (40 g/d) and creatine (3 g/d) in a male cohort representative of the North American aging community in general, e.g., individuals with low PA levels (sedentary/‘low active’), normal-to-high BMIs, and varying degrees of age-related muscle loss. We hypothesized that twelve weeks of HBRE would be beneficial for all participants, while the Muscle5 supplement would improve muscle integrity to a greater extent versus placebo.

## 2. Materials and Methods

### 2.1. Ethics

All methods and procedures in this randomized, double-blind, placebo-controlled trial were approved by the Hamilton Integrated Research Ethics Board (2018-4656-GRA). Study participants were informed about the potential risks of participation prior to giving their informed consent. This clinical trial was registered at clinicaltrials.gov (NCT03536871).

The current RCT was also part of a larger research project and collaboration between McMaster University (MAT), Exerkine Corporation (MAT), and Buck Institute for Research on Aging (Simon Melov, Novato, CA, USA) for evaluation of biomarkers of aging and muscle loss across different levels of sarcopenia. Specifically, some of the blood and muscle samples obtained at baseline from the participants were used for biomarker screening (muscle transcriptomics and serum proteomic analysis). Descriptive data and specific test outcomes, such as appendicular lean mass, maximal strength, and functional test results at baseline, were also shared between projects for characterization of subjects. The participants in the current RCT represent ~ 50% of the total number of samples in the sarcopenia biomarker project.

### 2.2. Participants

#### 2.2.1. Recruitment, Screening, and Randomization

**Figure 1:**
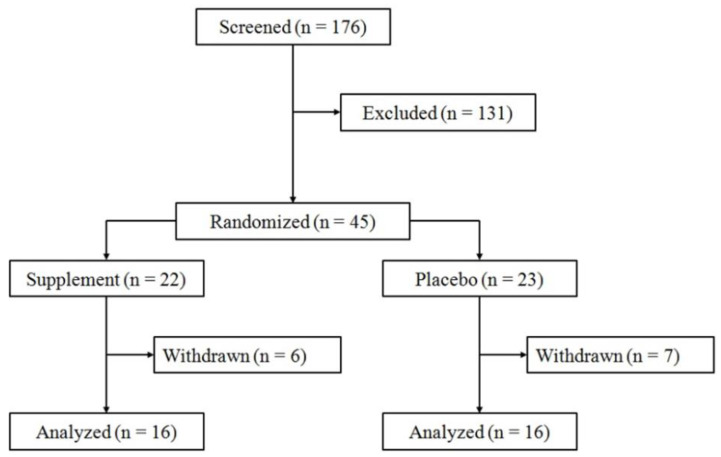
Flow diagram.

For the current RCT, we aimed to recruit older males representative of the majority of free-living (non-institutionalized and independent) elderly in North America, i.e., sedentary/’low active’, overweight/mildly obese, potentially polymorbid, and with varying degrees of muscle loss [73,74,75,76]. Secondly, because of the scarcity of data on the effects of HBRE and MIS in sarcopenic populations, we wanted to perform a sub-group analysis on those that were clinically confirmed as sarcopenic according to EWGSOP criteria [2]. All participants were recruited from the Greater Hamilton Area in Canada via local newspapers and poster advertisements in retirement homes, coffee shops, supermarkets, and veteran organizations. Media advertisements were most effective in garnering interest for the study and a total of 176 individuals were screened from May, 2018, to January, 2019 (Figure 1).

Potential participants were asked to complete an initial screen online or by phone to ensure they were of male gender, ≥65 years of age, free-living, non-smoking, sedentary or ‘low active’ (<150 min PA per week), and of normal to class 1/low-risk obesity according to body mass index (BMI; 20–34.9). Eligible individuals were then invited for an initial visit where they provided their written consent, medical/PA histories, and underwent an initial health screen to confirm eligibility (e.g., anthropometrics, body composition, and vitals).

Sample size calculations were based on the results of our previous study on the effects of MIS and RE on muscle integrity in older adults [67], setting power to 80% with alpha at 5%, yielding an estimate of 10 participants per group to detect differences in body composition and strength. Expecting a dropout rate of 20–30% for unsupervised training forms with limited subject interaction [36], and considering that sample sizes were 25 per group in Bell et al. [53], we aimed for a final count of 15–20 participants per group.

Out of 176 potential participants who completed the initial screening questionnaire, we enrolled 45 eligible older males, who completed a three-day diet log and a weekly step count to determine baseline macronutrient intake and PA levels prior to randomization. The subjects were then matched according to their baseline test results and randomly assigned to either Placebo (PLA; *n* = 23) or Muscle5 (M5; *n* = 22) by an independent third party not involved in subject recruitment, training, testing, nor having a financial stake in the company. Specifically, appendicular lean mass (ASM/H^2^), 1-RM grip strength, gait speed, age, bodyweight, protein intake, and step counts were matched as close as possible prior to randomization into PLA vs. M5 conditions (Table 1).

The 12-week intervention period was staggered between subjects with the first person commencing the multi-component therapy in June, 2018, and the last person ending their trial in April, 2019. Accounting for withdrawals and dropouts (29%; 13 out of 45), 32 participants completed pre- and post-testing in the current RCT (PLA; *n* = 16, M5; *n* = 16), in line with previous research on unsupervised training forms with limited subject interaction [36].

#### 2.2.2. Exclusion Criteria

Medical conditions that precluded participation were diabetes mellitus (requiring more than one anti-diabetic drug), recent myocardial infarction (<6 months ago), hypertension (requiring more than two medications), congestive heart failure (requiring more than one medication), previous stroke with residual hemiparesis, renal disease (creatinine > 140), liver disease, musculoskeletal injury affecting exercise tolerance, musculoskeletal disorder (other than age-related SM wasting), severe osteoporosis, severe osteoarthritis, severe peripheral neuropathy, chronic obstructive pulmonary disease (FVC or FEV1 <70% of age-predicted mean value), asthma (requiring more than two medications), gastrointestinal disease, infectious disease, inability to consent, lactose intolerance/dairy protein allergy, and the use of medications affecting protein metabolism (for example, corticosteroids). Lifestyle-associated behaviors that precluded enrollment were smoking, veganism, recent weight loss or gain (<3-month period prior to the study), PA levels exceeding the minimal recommendations (150 min/week), and intake of supplements that affect musculoskeletal metabolism (e.g., whey, casein, calcium, creatine monohydrate, vitamin D, and omega-3 fatty acids).

### 2.3. Experimental Design

**Figure 2:**
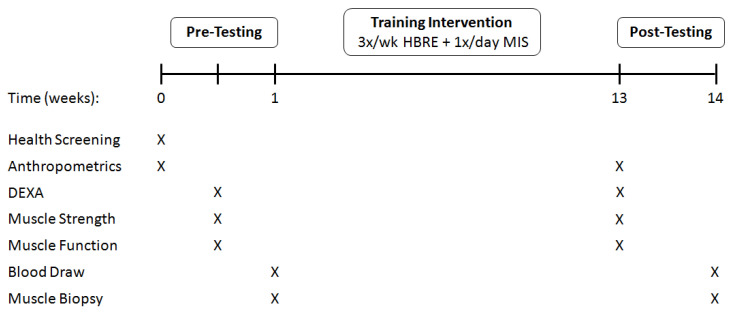
Experimental design.

The 12-week multi-component therapy consisted of 3/d week home-based, whole-body elastic band RE (HBRE) and daily intake of a protein-based 5-ingredient supplement (Muscle5, M5) or isocaloric/isonitrogenous placebo (Figure 2). Assessments of vital signs (HR and BP), anthropometry (weight and height), body composition (BMI, waist-to-hip ratio, and dual X-ray absorptiometry), function/performance (Short Physical Performance Battery, 6-M Gait Speed, Timed Up and Go, and 4-Step Stair Climb), strength (max grip, 1-RM leg press, and isometric knee extension), step-count (accelerometer), and dietary intake (3-day food record) were done one week prior to and following the interventional period. Blood samples and muscle biopsies were obtained pre and post study for biochemical analyses. Subjects arrived at the clinic in the fasted state (10–12 h) at the same time (AM) for both testing occasions. All procedures and tests were executed by the same nurse, doctor, and research technicians to minimize methodological variation and to ensure consistency within and between subjects.

### 2.4. Five-Ingredient Nutritional Supplement-Muscle5 (M5)

The “Muscle 5” supplement (M5) was so termed due to the fact that it contains five nutrients generally regarded as safe (GRAS) that are available in most food, drug, and health food retailers in North America, specifically, protein (whey (24 g/d), micellar casein (16 g/d)), creatine (3 g/d), vitamin D3 (1000 IU/d), and omega-3 containing fish-oil (EPA; 1.51 g/d, DHA; 0.95 g/d) (Table 2). Calcium (416 mg/d) was contained naturally within the micellar casein. Sucrose, stevia, and chocolate flavor were included as flavoring agents in both PLA (e.g., collagen and sunflower oil) and M5, which were isocaloric (272 kcal per serving/day), isonitrogenous (40 g protein), and identical in flavor, smell, and appearance. All products were manufactured, blinded and provided by Infinit Nutrition (Windsor, ON, Canada), stored in sachets (dry products) or brown bottles (oils), and delivered to the subjects in a double-blind fashion with a coded numbering system. The allocation was not revealed to the investigators until the data were cross-referenced, locked, and simultaneously provided to Infinite Nutrition and three of the authors. The locked data and the decoding system are available upon request.

Participants were instructed to consume two teaspoons of oil (~10 mL) and one sachet of dry products with water (~350 mL) in the morning with breakfast throughout the 12-week intervention period, while maintaining their normal dietary habits. At-home compliance was checked by email or phone on a bi-weekly basis and the subjects were instructed to record their daily intake in a supplement log.

### 2.5. Home-Based Resistance Exercise (HBRE)

In addition to daily intake of either PLA or M5, participants were instructed to engage in whole-body, progressive RE with elastic bands (TheraBand, Hygenic Co., Akron, OH, USA) three times a week on non-consecutive days for 12 weeks. Specifically, the HBRE program consisted of six lower body and six upper body exercises, including biceps curl, triceps extension, lateral raise, seated row, bench press, abdominal crunch, calf raise, chair squat, knee extension, knee flexion, hip flexion, and dorsi flexion.

Exercises were to be performed in a controlled fashion with a focus on correct technique for 3 sets of 10–15 repetitions each, and elastic band tension force (i.e., resistance) was progressively increased throughout the study for continual adaptation (yellow, 1.32 kg; red, 1.77 kg; green, 2.27 kg; blue, 3.22 kg; and black, 4.40 kg [77]). Prior to starting the HBRE paradigm, subjects were shown in person and given a handout with clear instructions on technique and progression (https://www.youtube.com/watch?v=ZicHgR4NNS4), which included a set of stretching exercises to be performed as warm-up and cool-down. Finally, participants were instructed to walk at least 5000 steps on exercise days and 10,000 steps on rest days. At-home compliance was checked by email or phone on a bi-weekly basis and the subjects were instructed to record their RE and steps in an exercise log.

### 2.6. Anthropometry, Vitals, and Body Composition

Several anthropometric and vital measures were obtained pre and post study, including body weight (Health O Meter Professional, McCook, IL, USA), height (Perspective Enterprises, Portage, MI, USA), body mass index (BMI; kg/m^2^), heart rate, and arterial blood pressure (Marshall Nurse Stethoscope, Riverside, IL, USA; MDF Instruments Sphygmomanometer, Agoura Hills, CA, USA).

### 2.7. Sarcopenia Diagnosis

As per the 2010 recommendations by the European Working Group on Sarcopenia (EWGSOP) [2], sarcopenia was classified in a three-pronged process that included tests for muscle mass, strength, and physical performance (see below). Severity of age-related muscle loss was characterized as pre-sarcopenia (low muscle mass only), sarcopenia (low muscle mass and strength and/or performance), or severe sarcopenia (low muscle mass, strength and performance). Baseline diagnostic test results, such as ASM/H^2^, 1-RM grip strength and gait speed, were reasonably matched between PLA and M5 groups prior to randomization.

### 2.8. Body Composition Testing

In order to assess the first diagnostic domain of sarcopenia (e.g., muscle mass), body composition was measured pre and post study using dual X-ray absorptiometry (DXA; GE Lunar Prodigy, Madison, WI) with a software program for adults (ver. enCORE 9.15.010), including whole body lean mass (total lean mass; TLM), regional lean mass (appendicular lean mass; ASM), absolute and relative fat mass, and bone mineral density. As previously described, the cut-off point for sarcopenia was ≤7.26 kg/m^2^ for ASM/h^2^, using the original EWGSOP guidelines from 2010 rather than 2019 updates [2,3].

All scans were analyzed in batches by a trained technician blinded to the experimental conditions. Body positions, bony landmarks, and scan table references were kept constant and ROIs were matched between each individual’s pre- and post-intervention scans. Spine phantom quality control scans were performed first thing in the morning and as needed to ensure machine consistency and account for any significant variability in performance.

### 2.9. Maximal Strength Testing

The second diagnostic domain of sarcopenia involves testing for muscle strength [2,3]. In order to assess upper and lower body strength improvements, subjects were asked to perform a battery of maximal exertion tests pre and post intervention. Strength testing consisted of 1-RM grip strength, 1-RM leg press, and 1-RM isometric leg extension tests, which were chosen because they are correlated to physical impairment, morbidity, and mortality in older adults [78]. While the lower limbs are more relevant to gait speed and leg strength [79], 1-RM grip strength is a well-established ‘biomarker of aging’, predicts cause-specific mortality, has clear cut-off points for clinically significant muscle weakness, and is recommended by EWGSOP for sarcopenia diagnosis [2,3,80]. Notably, the updated recommendations by EWGSOP suggest that muscle strength should be the principal determinant of sarcopenia since it is a better predictor of adverse outcomes than muscle mass [3]. For the current study, the cut-off point was ≤30 kg for 1-RM grip strength in males, consistent with the original EWGSOP guidelines [2].

For the 1-RM grip strength test, the subjects performed three maximal repetitions with their dominant hand using a hand grip dynamometer (Jamar; Lafayette Instrument Company, Lafayette, IN, USA) in the seated position according to the Southampton protocol [81,82]. Secondly, the 1-RM leg press test was done using a traditional weight stack machine (Cybex Eagle), with the subjects increasing the resistance by 5–10 kg until reaching maximum or “very, very hard” on the Borg scale. Maximal isometric knee extension (peak torque) was obtained using a Biodex dynamometer (System 3, Biodex Medical Systems Inc., Shirley, NY, USA) as described previously [67,78]. Briefly, subjects were positioned into the Biodex machine with their knees flexed at 90 degrees while performing 3 × 5 s isometric contractions with 30 s of rest between each attempt.

All tests were demonstrated, supervised, and recorded by the same technicians on pre- and post-intervention visits. Subjects were familiarized with the equipment and allowed a warm-up prior to any testing. Maximal attempts were completed with verbal encouragement and only the highest values were used for data analyses.

### 2.10. Performance Testing

The last diagnostic domain of sarcopenia involves assessing performance limitations of the lower extremities. A wide range of tests were administered on pre- and post-intervention visits, including the Short Physical Performance Battery (SPPB), which has defined cut-offs for increased fall risk, frailty, disability, and sarcopenia [2,3,83,84]. The multi-component SPPB specifically measures 4-M Gait Speed, 5-Times Sit-to-Stand time (“chair stand”), and balance (feet together, semi tandem, and full tandem), with each component scored from 0 to 4 and summed for a total extremity function/mobility limitation score (0–3, severe; 4–6, moderate; 7–9, mild; and 10–12, minimal) [84]. Lastly, complementary to the SPPB, 6-M Gait Speed, Timed Up and Go (TUG), and 4-Step Stair Climb performance were assessed as previously described [3,85,86,87]. A score lower than 8 for SPPB, >15 s for chair stand, ≤0.8 m/s for gait speed, ≥20 s for TUG, and ≥two standard deviations from a young reference group (data available upon request) for stair climb were used as cut-off points for sarcopenia [2,3]. All tests were administered by the same technicians and standardized between pre and posts visits.

### 2.11. Muscle Quality

As previously described, muscle quality (MQ) is an indicator of muscle function defined as strength per unit muscle [79]. MQ is calculated by dividing upper body or lower body strength by lean mass of arms or legs, respectively, and has previously been used in sarcopenia RCTs [77]. In this study, we calculated MQ by dividing both upper and lower body strength (hand grip + ([leg press + leg extension]/2)) by appendicular lean mass.

### 2.12. Physical Activity

As a part of the initial screening process, a physical activity (PA) questionnaire was administered to all potential participants for estimation of work, sports, and leisure activities [88]. A detailed description of past and current PA levels was also provided by the subjects prior to study participation. The subjects’ step counts were then measured with a standard accelerometer (Omron HJ-321 Alvita Pedometer) to match PA levels between PLA and M5 groups as close as possible at baseline. Participants were also asked to keep a daily record of their steps and other activities in a log to monitor PA levels during the course of the study.

### 2.13. Dietary Records

All participants kept a detailed food record for three non-consecutive days (e.g., two weekdays and either a Saturday or a Sunday) one week prior to the start and at the end of the trial. The food records were analyzed using the ASA-24 software (National Cancer Institute) to obtain caloric, macronutrient, vitamin, and mineral intake, including carbohydrate, protein, fat, vitamin D3, calcium, and n-3 PUFAs. Protein intake, specifically, was closely matched between PLA and M5 groups prior to randomization. Subjects were encouraged to maintain their regular food intake and record any changes to their consumption during the course of the trial.

### 2.14. Muscle Biopsies

As previously described, percutaneous needle muscle biopsies were obtained pre and post 12-week intervention from the vastus lateralis muscle under local anesthesia (2% xylocaine) with the use of a 5-mm Bergstrom needle modified for manual suction [89]. Muscle samples were dissected free of any visible fat and connective tissue, and 25 mg of muscle was embedded in optimal cutting temperature medium (OCT) and stored at −80 °C until cryosectioned.

### 2.15. Immunofluorescence

OCT-embedded samples were cryosectioned to obtain 8 µm thick sections and mounted on glass slides for immunofluorescence detection of myosin heavy chains (MHC) [90]. Briefly, slides were rinsed in PBS, then blocked with 10% goat serum in 1% BSA-PBS and incubated overnight at 4 °C with the following primary antibodies: BA-F8 (MHC I; Developmental Studies Hybridoma Bank (DSHB), Iowa City, IA, USA; 1:100), SC-71 (MHC IIA/IIX; DHSB; 1:600), and 6H1 (MHC IIX; DHSB; 1:25). Following incubation, slides were rinsed in 1X PBS and the appropriate secondary antibodies were applied for 1 h (Alexa Fluor 488, 555 or 647, Thermo Fisher Scientific). Slides were once again washed in 1× PBS and Wheat Germ Agglutinin (Alexa Fluor 350 conjugate, Thermo Fisher Scientific, 1:300) was applied for 15 min. Lastly, slides were washed in 1× PBS and coverslips were mounted using fluorescence mounting medium (Dako, Burlington, ON, Canada) and muscle cross-sections were imaged using a CoolSNAP HQ2 fluorescence camera at 20 times magnification (Nikon Instruments, Melville, NY, USA) (Figure S1). Pre and post time-points for each subject were sectioned, stained and imaged on the same slide and day.

### 2.16. Muscle Fiber Analysis

All muscle samples containing ≥ 200 fibers were analyzed for fiber type composition (MHCI, MHCIIA and MHCIIX) and cross-sectional areas (CSAs). On average, 50 fibers per fiber type were measured using the Nikon NIS Elements AR 4.40 software (Nikon Instruments). If the muscle sample contained less than 25 fibers for a certain fiber type, the CSA data for that fiber type was removed from the analysis for both pre and post time points. All measures were done in duplicate by two independent researchers, who were blinded to the conditions, and thereafter averaged for the final data analysis.

### 2.17. Blood Sampling and Analyses

All blood samples were drawn from the antecubital vein into anticoagulant-treated (heparin; plasma) or untreated (serum), evacuated tubes using venipuncture kits and sterile precautions. Serum was allowed to clot for 1 h at room temperature prior to processing, while plasma samples were mixed by inversion and then processed. Standard centrifugation at 2000× *g* for 15 min at 4 °C was done to separate cells from supernatants, with the latter retained for biochemical analyses of liver and kidney function, inflammation, and blood lipids by the Core Laboratory at Hamilton Health Sciences Centre (Ontario Laboratory Accreditation Certified). Creatinine, bilirubin, alanine aminotransferase (ALT), gamma glutamyltransferase (GGT), C-reactive protein (CRP), low density lipoprotein (LDL), high density lipoprotein (HDL), total cholesterol, and triglycerides were assessed on all subjects and time points (pre and post).

### 2.18. Statistical Analyses

As previously described by Gupta et al. [91], a better application of the intention-to-treat (ITT) approach is to only include those randomized participants with complete outcome data, excluding dropouts and/or those with major protocol violations (i.e., ‘per protocol population’). Therefore, thirteen randomized subjects, who did not initiate or complete the 12-week multi-component therapy, were excluded from the final statistical analyses (Figure 1).

Co-primary outcomes for this RCT were fat-free mass/total lean mass (TLM) and lower body strength (leg press), based upon the main outcomes from an earlier RCT using a similar blend of nutrients combined with supervised RE [53]. The secondary outcomes were appendicular lean mass (ASM and ASM/H^2^), isometric knee extension and grip strength, muscle/body fat ratios (TLM/% Body Fat and ASM/% Body Fat), muscle fiber cross sectional areas (Type I, Type IIa and Type IIx CSAs), and function/performance outcomes; Timed Up and Go (TUG), 4-M Gait Speed, 5-Times Sit-to-Stand, 4-Step Stair Climb, and Short Physical Performance Battery (SPPB).

Longitudinal adaptations (e.g., ∆ Pre vs. Post main effect of ‘Time’) within each experimental condition were of primary interest. As such, ‘Time’ (Pre vs. Post), ‘Treatment’ (HBRE and PLA vs. HBRE and M5), and interactive (‘Time × Treatment’) effects were analyzed by two-way repeated measures ANOVA, followed by LSD post hoc testing if either of the omnibus F-tests were significant. A planned sub-group analysis of the longitudinal effects of the multi-component therapy in sarcopenic individuals was also administered using paired *t*-tests. All statistical analyses were done with the Statistica program (STATISTICA Version 8.0; StatSoft, Inc.; Tulsa, OK, USA). Significance was set at *p* < 0.05, and data are presented as means ± standard error (SE).

## 3. Results

### 3.1. Withdrawals, Dropouts, and Compliance

In this trial, 45 individuals of European origin (Caucasian decent) were randomized into either PLA or M5 groups. Thirty-two out of 45 participants finished the full 12-week intervention (Figure 1). Three individuals withdrew prior to the start of the study, while 10 dropped out during the intervention, with an even distribution across groups, consistent with previous reports on unsupervised, long-term RE interventions with limited subject interaction [36]. Reasons for withdrawal/dropout included unrelated surgery (*n* = 2), unrelated health issues (*n* = 2), muscle soreness (*n* = 1), travel (*n* = 2), flavor of oil (*n* = 1), time constraints (*n* = 1), and other personal reasons (*n* = 4).

Compliance to the home-based, multi-component therapy was comparable to those in supervised MIS/RET trials [53], with no significant differences in adherence to supplementation (95.4 ± 1.9% vs. 89.3 ± 5.0%) or HBRE (89.1 ± 8.1% vs. 84.1 ± 6.1%) between PLA and M5 groups, respectively (Table 3).

### 3.2. Macronutrient Intake and Daily Physical Activity

Of primary relevance to sarcopenia, daily protein intake was not different between PLA (0.97 ± 0.07 g/kg BW) and M5 (1.07 ± 0.12 g/kg BW) groups at baseline, which was also the case for other macronutrients and total caloric intake (Table 3). As expected, total protein consumption, adjusted for supplementation and compliance, was significantly higher in PLA (1.51 ± 0.08 g/kg BW) and M5 (1.34 ± 0.08 g/kg BW) groups post intervention and exceeded the current RDA (0.8 g/kg BW) but also in line with optimal protein intake for SM maintenance in elderly (≥1.2 g/kg BW/d) [56,57,58]. However, it is important to note that the anabolic potential of whey (WPI 895-I) and micellar casein (MPI 4900), which made up 35% of total protein intake in the M5 group, is higher than collagen in the PLA group. Daily intakes of vitamin D3, calcium, and n-3 PUFAs were significantly lower than RDAs in both groups at baseline, but significantly higher in the M5 group post study. Importantly, all post study intakes remained within the upper safety limits established by Health Canada (https://www.canada.ca/en/health-canada/services/food-nutrition.html).

Physical activity levels, as measured by step counts, were not different between PLA (Pre: 5899 ± 772 and Post: 5230 ± 483) or M5 (Pre: 5835 ± 808 and Post: 5659 ± 592) groups, nor did they change post study (Table 3). The average step counts were within the normal range for this population (2000–9000 steps) [92], while they were technically classified as ‘low active’ and therefore in line with our recruitment strategy.

### 3.3. Anthropometry, Vitals, and Descriptives

As per the norm in North America, the participants were on average overweight/mildly obese, hypertensive (stage 1/medium risk), prediabetic, and used multiple medications (Table 1) [73,74]. Specifically, 11 individuals were classified as obese, and the remainder were either normal (*n* = 6) or overweight (*n* = 15) according to body mass index.

Following 12 weeks of intervention, body weights and BMIs were generally increased (main effect of ‘Time’; *p* ≤ 0.05), but only reached statistical significance in placebo-treated individuals. The multi-component therapy had no other apparent effects on anthropometry, vital measures, or glycemia.

### 3.4. Body Composition and Muscle Mass

**Figure 3:**
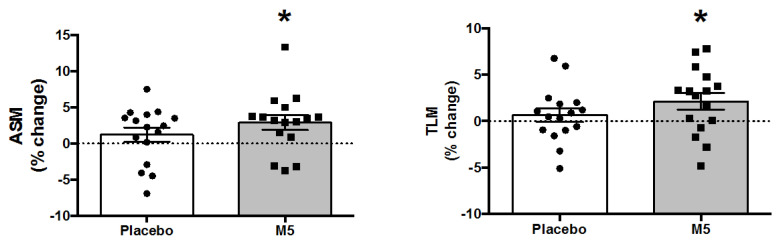
Percent changes in lean mass following 12 weeks of multi-component therapy (HBRE/M5) in old males. Abbreviations: M5; ‘Muscle5’ Multi-Ingredient Supplement, HBRE; Home-Based Resistance Exercise, ASM; Appendicular Lean Mass, TLM; Total Lean Mass. * Significantly increased vs. baseline by two-way repeated measures ANOVA (*p* ≤ 0.05). *Y*-axis value 0 = baseline. Absolute values are shown in Table 1.

**Figure 4:**
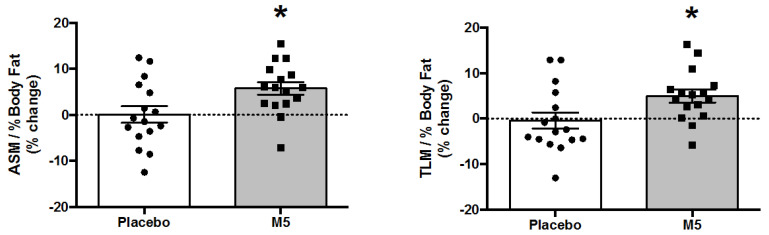
Percent changes in lean mass-to-body fat ratios following 12 weeks of multi-component therapy (HBRE/M5) in old males. Abbreviations: M5; ‘Muscle5’ Multi-Ingredient Supplement, HBRE; Home-Based Resistance Exercise, ASM; Appendicular Lean Mass, TLM; Total Lean Mass. * Significantly higher vs. baseline by two-way repeated measures ANOVA (*p* ≤ 0.05). *Y*-axis value 0 = baseline. Absolute values are shown in Table 1.

Total and appendicular lean mass were generally improved in participants following the intervention, with a main effect of ‘Time’ for TLM (*p* = 0.039), ASM (*p* = 0.009), and ASM/h^2^ (*p* = 0.009) (Table 1). Post hoc testing revealed that the improvements were only statistically significant for the M5 group (Figure 3), with an average gain of 1.09 kg (TLM) and 0.69 kg (ASM), which is comparable to other MIS/RE RCTs [55]. In addition, because subjects receiving the placebo gained body fat during the course of the trial, while those on M5 lost body fat, there was a significant ‘Time × Treatment’ interaction on BF percentage (*p* = 0.012). Therefore, ‘Time × Treatment’ interactions on muscle-to-body fat ratios, specifically ASM/BF Mass (*p* = 0.028), TLM/% BF (*p* = 0.017), and ASM/% BF (*p* = 0.02), were also significant and thus driven by improvements in overall body composition in the M5 group (Figure 4).

Sub-group analysis revealed that sarcopenic individuals in both M5 (‘Sarcopenic M5’) and PLA (‘Sarcopenic Placebo’) groups exhibited significant gains in appendicular lean mass post intervention (*p* ≤ 0.05), with an average gain of 0.9 kg (‘Sarcopenic All’) (Table S1). Total lean mass (∆ Pre–Post TLM; +1.65 kg) and body fat (∆ Pre–Post BF; −0.7 kg, *p* = 0.058, ∆ Pre–Post %BF, −1.3%, *p* < 0.05) were only improved in the M5 group (*p* < 0.05). As such, consistent with the main findings, those receiving the M5 gained lean mass (ASM and TLM) and lost body fat, significantly improving all muscle-to-body fat ratios (*p* < 0.05). Collectively, four out of seven sarcopenic individuals were reclassified into less severe sarcopenia stages, or even ‘normal’, following the intervention.

### 3.5. Muscle Fiber Cross-Sectional Areas

**Figure 5:**
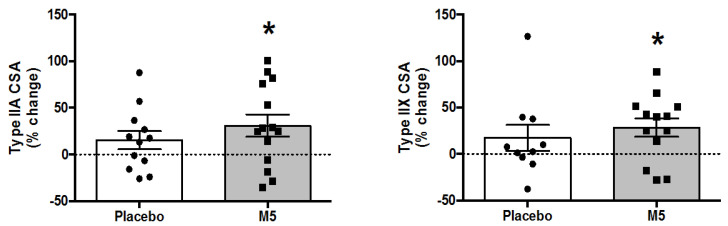
Percent changes in muscle fiber cross-sectional areas (CSAs) following 12 weeks of multi-component therapy (HBRE/M5) in old males. Abbreviations: M5; ‘Muscle5’ Multi-Ingredient Supplement, HBRE; Home-Based Resistance Exercise, CSAs; muscle fiber cross-sectional areas. * Significantly larger vs. baseline by two-way repeated measures ANOVA (*p* ≤ 0.05). *Y*-axis value 0 = baseline. Absolute values are shown in Table 4.

We observed general hypertrophy of type II muscle fibers, and to a lesser extent Type I muscle fibers, following the study (Table 4). Repeated measures ANOVA revealed a main effect of ‘Time’ on Type IIa (*p* = 0.006) and Type IIx (*p* = 0.018) muscle fiber cross-sectional areas (CSAs), while Pre–Post improvements were only significant in the M5 group (%∆ CSAs; Type IIa +30.9 ± 11.8%, Type IIx +28.5 ± 9.9%) (Figure 5). Fiber type distributions remained relatively stable between time points, although there was a significant ‘Time x Treatment’ (*p* = 0.037) interaction for Type IIx CSAs.

Consistent with the main findings, we observed general hypertrophy of Type IIa (%∆ CSA; +23 ± 7.9%, *p* = 0.029) and Type IIx (%∆ CSA; +19 ± 7.3%, *p* = 0.062) fibers in sarcopenic individuals (Table S1). Additionally, average improvements appeared to be more pronounced in the M5 (%∆ CSA; Type I +30 ± 39%, Type IIa +37 ± 8.1%, Type IIx +33 ± 6.3%) vs. PLA group (%∆ CSA; Type I −1.5 ± 12.9%, Type IIa +10 ± 8.3%, Type IIx +5 ± 2.6%), although statistical power was too low to reach significance.

### 3.6. Muscle Strength

**Figure 6:**
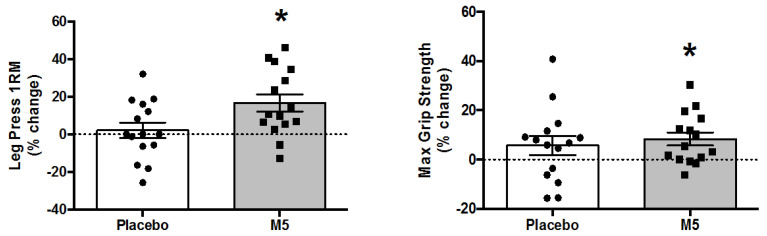
Percent changes in upper and lower body maximal strength following 12 weeks of multi-component therapy (HBRE/M5) in old males. Abbreviations: M5; ‘Muscle5’ Multi-Ingredient Supplement, HBRE; Home-Based Resistance Exercise. * Significantly increased vs. baseline by two-way repeated measures ANOVA (*p* ≤ 0.05). *Y*-axis value 0 = baseline. Absolute values are shown in Table 1.

Self-reported data indicated that TheraBand resistance was increased from 2.06 ± 0.1 kg at baseline to 2.83 ± 0.09 kg post study (% ∆ Pre–Post; +37%) collectively as per the progressive overload principle, although there was no significant difference in endpoint elastic band tension force between groups (data not shown). Maximal strength was generally improved in all participants, with a main effect of ‘Time’ for leg press (*p* = 0.011) and hand grip (*p* ≤ 0.01) and borderline significance for knee extensor strength (*p* = 0.064) (Table 1). However, post hoc analyses were only significant for the M5 group, including leg press (% ∆ Pre–Post; +16.6 ± 4.6%) and hand grip (% ∆ Pre–Post; +8.4 ± 2.7%) (Figure 6) but not knee extensor strength (% ∆ Pre–Post; +6.8 ± 3.9%).

Interestingly, improvements in maximal strength appeared to be more robust in sarcopenic vs. non-sarcopenic individuals, including leg press (% ∆ Pre–Post; +25%, *p* = 0.13), hand grip (% ∆ Pre–Post; +9%, *p* = 0.07), and knee extension (∆ Pre–Post; +27%, *p* = 0.057) in the M5 group, although Pre–Post tests were not statistically different (Table S1).

### 3.7. Performance Tests

**Figure 7:**
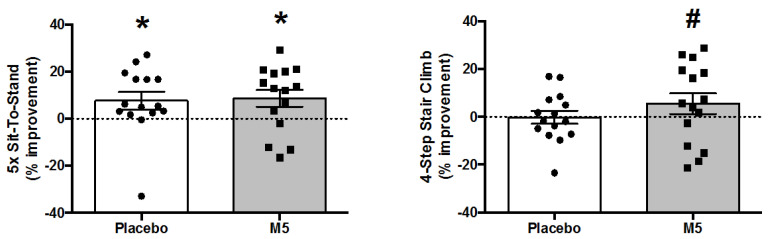
Percent changes in performance following 12 weeks of multi-component therapy (HBRE/M5) in old males. Abbreviations: M5; ‘Muscle5’ Multi-Ingredient Supplement, HBRE; Home-Based Resistance Exercise. * Significantly increased vs. baseline by two-way repeated measures ANOVA (*p* ≤ 0.05). # Significantly higher vs. baseline by paired *t*-test (*p* ≤ 0.05). *Y*-axis value 0 = baseline. Absolute values are shown in Table 1.

Post study performances were generally improved across all tests as compared to baseline, although statistical significance was only observed in the 5-Times Sit-to-Stand test (e.g., main effect of ‘Time’; *p* ≤ 0.01) (Table 1). Post hoc analysis revealed that both PLA and M5 groups performed the test faster following the intervention (*p* < 0.05), thus confirming the efficacy of home-based, low-intensity RE to improve muscle function in older adults (Figure 7). While there were no other significant main effects or interactions for performance tests by 2 × 2 ANOVA, 4-Step Stair Climb time was significantly improved in the M5/HBRE group when analyzed separately by paired *t*-test (*p* < 0.05) (Figure 7).

Consistent with the strength outcomes, the magnitude of improvements for performance tests appeared more robust in sarcopenic vs. non-sarcopenic individuals, with significant Pre–Post differences for 4-M Gait Speed (*p* = 0.013), 6-M Gait Speed (*p* = 0.017) and 4-Step Stair Climb (*p* = 0.047), but not for TUG (*p* = 0.18), SPPB (*p* = 0.14) or 5-Times Sit-to-Stand (*p* = 0.083) (Table S1; ‘Sarcopenia All’). Importantly, when analyzing the PLA and M5 groups separately, improvements were only significant for the 6-M Gait Speed and 4-Step Stair Climb tests in the M5 group.

### 3.8. Muscle Quality

**Figure 8:**
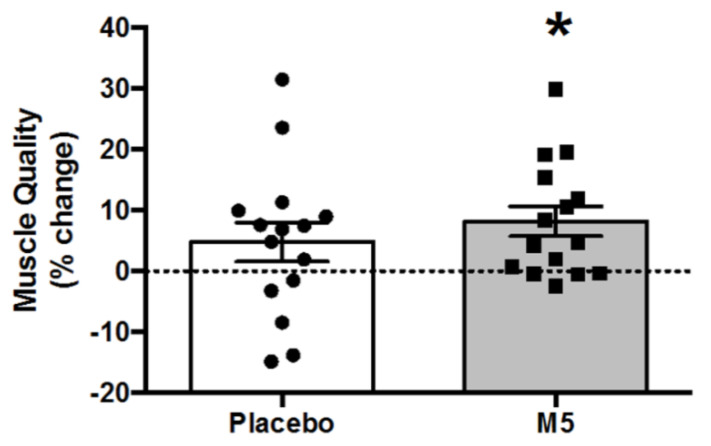
Percent changes in muscle quality (MQ) following 12 weeks of multi-component therapy (HBRE/M5) in old males. Abbreviations: M5; ‘Muscle5’ Multi-Ingredient Supplement, HBRE; Home-Based Resistance Exercise. * Significantly improved vs. baseline by two-way repeated measures ANOVA (*p* ≤ 0.05). *Y*-axis value 0 = baseline. Absolute values are shown in Table 1.

Muscle quality (MQ) was significantly improved in the M5/HBRE group only (Figure 8; *p* < 0.01), indicative of an overall enhanced muscle function. MQ was similarly improved in sarcopenic individuals although this did not reach statistical significance (Table S1).

### 3.9. Blood

Twelve weeks of M5/HBRE therapy had no significant or unexpected effects on markers of liver function, inflammation or lipid profiles (Table 5). Although serum creatinine levels were altered post intervention in the M5 group, higher muscle mass and creatine supplementation (e.g., increased rate of appearance not reduced renal clearance) will naturally elevate creatinine levels without underlying kidney dysfunction [93]. As previously discussed by others, commonly used estimates of kidney function that are based on serum creatinine levels must be interpreted in the broader context of total protein intake and creatine supplementation [94]. The participants in this trial had no pre-existing liver or kidney disease and creatine is currently considered safe for aging populations [95]. Additionally, the daily dosage in the current RCT (3 g/d) is at the lower end of the spectrum compared to previous trials on creatine supplementation [95].

## 4. Discussion

In this 12-week randomized, placebo-controlled clinical trial, we tested the efficacy of daily intake of a five-ingredient supplement (e.g., whey, micellar casein, creatine, vitamin D3, and n-3 PUFAs (‘Muscle5’; M5)), combined with three days per week low-intensity HBRE, for improving SM mass, strength, and function in free-living elderly. A similar blend of ingredients, although at a significantly higher dosage, was previously shown to potentiate SM gains in a homogenous group of healthy, older men independent of and in conjunction with supervised RE/HIIT [53]. We now report that M5/HBRE therapy enhances lean mass, strength, muscle-to-fat ratios, muscle quality and Type II fiber growth in older men with low physical activity levels, wide BMI range, moderate health issues, and with varying degrees of age-related muscle loss. Our sub-group analysis further suggests that M5/HBRE may be beneficial for mitigating sarcopenia, as the magnitude of improvements appeared to be greater in sarcopenic vs. healthy individuals. Importantly, the current M5 supplement provided similar muscle benefits compared to the previous iteration [53], in spite of it containing 33% less total protein and 40% less creatine for improved gastrointestinal tolerability, total bulk density and to fit within national health product guidelines for Canada (https://www.canada.ca/en/health-canada/services/drugs-health-products/natural-non-prescription.html). It is also important to note that the benefits of the current M5 product were observed in conjunction with an unsupervised, home-based exercise program as compared to the earlier iteration with a gym-based, supervised program. Given the recent experience with a near global lock-down of all gymnasia and health clubs from the COVID-19 pandemic, this latter aspect of the study provides for immediate practical application to the general population and, in particular, the elderly.

Biological aging is inevitable and the etiology of sarcopenia is complex (for general reviews see [96,97,98,99]), multi-factorial, and involves dysfunction of all four basic tissue types, including nerve, muscle, connective, and epithelial tissues. Notwithstanding, skeletal muscle growth is ultimately governed by the balance between the immediate growth regulatory processes within skeletal muscle, specifically protein turnover (synthesis and degradation), satellite cell donation, and cell death. Documented age-deficits in these processes include anabolic resistance to insulin receptor stimulation (blunted Akt-mTOR signaling, eukaryotic translation initiation, and MPS) [41,42,100], reduced insulin receptor-mediated inhibition of proteolysis [101], satellite cell dysfunction [102,103,104], and activation of apoptosis [105,106]. These age-related decrements typically impair the growth response to standard exercise and nutritional monotherapies and the idea of using a multi-component therapy (for example, exercise and nutrition) rests on simultaneous targeting of several pathways to potentiate SM growth.

The same logic applies to MIS vs. single-nutrient supplementation as the former theoretically stimulates several processes simultaneously, thus ‘circumventing’ the main limitation of using nutritional monotherapies, such as whey or BCAAs, e.g., anabolic resistance with aging. Common nutrients with documented SM benefits in MIS include whey [53,69,70], vitamin D [53,61,62,69,70], polyunsaturated fatty acids (n-3 PUFAs; EPA and DHA [53,63,64,69,70]), and creatine [53,65,66,67,68,69,70]. As previously demonstrated by Bell et al., this blend of nutrients may be safely combined to potentiate SM growth independently or in conjunction with supervised RE/HIIT [53]. The current M5/HBRE trial confirmed this observation in a heterogenous group of individuals with a lower daily dosage for total protein, whey, and creatine, but with added casein as a complement to whey. Importantly, these common MIS ingredients appear to have benefits beyond the musculoskeletal system, including improved cognition, higher n-3 index, and anti-inflammatory effects in older adults [69,70]. Thus, the observation that protein monotherapy may be as effective as protein-based MIS for SM growth [55] appears to be of mostly academic interest and less applicable to maintaining overall health at old age.

In the current RCT, 12 weeks of M5/HBRE therapy synergistically enhanced lean mass and maximal strength (e.g., co-primary outcomes), which ultimately improved overall muscle quality (MQ) of the participants. The observed gains are comparable to or even greater than those reported by O’Bryan et al. in their recent meta-analysis on 35 MIS and supervised RE trials. On average, long-term MIS/RE (e.g., ≥ six weeks) appears to increase TLM by +0.8 kg (95% CI 0.44–1.15) and 1-RM upper and lower body strength by +2.56 kg (95% CI 0.79–4.33) and +4.22 kg (CI 0.79–7.64), respectively [55]. In this meta-analysis, untrained and older adults were found to exhibit even greater SM gains vs. trained or young individuals [55], which is consistent with our findings in that the most fragile participants responded favorably to M5/HBRE therapy even at the muscle fiber level. Importantly, the hypertrophic response in the current RCT (e.g., +28–37% Type II CSAs), whether in sarcopenic or non-sarcopenic individuals, is comparable to that of supervised RE trials of similar duration [29].

A significant proportion of elderly suffer from chronic musculoskeletal pain [107], necessitating low-impact RE forms, such as elastic band training (EBT), for decreased discomfort, reduced risk of injury, and improved adherence. Supervised EBT (without supplementation) has previously been shown to mitigate physical deconditioning and improve muscle performance in sarcopenically obese [77] and institutionalized [108] older women, respectively. De Liao et al. reported that 12 weeks of supervised EBT improved DXA-derived lean mass, overall body composition, muscle strength, and performance in subjects with sarcopenic obesity [77]. Compared to the current EBT trial, absolute gains in TLM and leg lean mass were lower, while strength and performance gains were greater than those in the placebo-treated, sarcopenic subgroup. Multi-nutrient supplements, including leucine, essential amino acids, and various vitamins, have also been tried with supervised EBT but with limited effectiveness beyond the exercise regimen itself [108,109]. Our results partly corroborate these findings in that home-based EBT provided modest improvements in muscle mass and strength in placebo-treated individuals, and equaled the efficacy of M5/HBRE therapy on specific performance tests (e.g., 5-Times Sit-to-Stand). However, as we only observed significant ∆ gains in muscle fiber CSAs, lean mass, strength and MQ in those receiving the M5 supplement, our results ultimately support the use of MIS with low-intensity HBRE for SM maintenance at old age.

Other important factors to consider for long-term adherence to exercise interventions are cost and time. Home-based interventions are generally less expensive, time-saving and flexible, and promote self-responsibility in the health care process. Obviously, these benefits are considerable, but must be weighed against efficacy, withdrawal/dropout rates, and protocol adherence. The efficacy of 12-weeks of supervised vs. home-based mixed training (aerobic, RE, balance, and gait training 2–3 d/week) on muscle mass, strength, and performance was recently investigated in sarcopenic individuals by Tsekoura et al. [34]. Twelve weeks of supervised training significantly improved 15/17 outcomes, compared to 7/17 for the home-based group, meanwhile limiting dropouts (0) and maintaining protocol adherence (87–92%) for both interventions. Our results are in general agreement with these findings and those of Maruya et al. [36], collectively demonstrating that home-based interventions provide significant muscle benefits to older adults with varying degrees of muscle loss, although to a lesser extent than supervised training forms. Adherences were comparable between studies, while dropout rates were significantly higher in Maruya et al. (23%) and our study (29%), potentially necessitating more frequent follow-ups and visits during home-based interventions.

At-home PA interventions and nutritional management have never been more applicable than during the COVID-19 (SARS-CoV-2) pandemic in 2020. While certain risk factors, such as old age and male gender, are unchangeable and known to be associated with a higher risk of developing severe COVID-19 complications [110], others may be modifiable, such as physical activity levels and nutritional intake. Most countries across the world have imposed self-isolation laws and guidelines for an extended period of time that has led to restricted access to parks, gyms, and sporting facilities. These restrictions are particularly relevant to high-risk populations, including elderly, immune-compromised, and those with poly-morbidity (CVD, diabetes, obesity, etc.) [110,111], considering they are already predisposed for aerobic deconditioning and wasting of skeletal muscle. The forced lockdown has already been shown to lead to lower PA levels in those over 55 y of age and in those who typically exercise in a gym setting [39]. A reduction in PA levels is now well-recognized to be one of the main factors associated with the development of obesity and diabetes, both of which are strong predictors of COVID-19 lethality [111]. Furthermore, a study of nearly 400,000 people in the United Kingdom found a higher risk of being admitted to the hospital with COVID-19 amongst those who were inactive (R.R. = 1.32, C.I. = 1.10, 1.58) and obese (R.R. = 2.05, C.I. = 1.68, 2.49) [112].

Specifically, low-to-moderate intensity exercise provides a hormetic stress stimulus that transiently activates evolutionary conserved cell danger response programs and, when repeated regularly, confers multi-systemic health benefits and protection against a spectrum of diseases [113], including respiratory tract infections and acute respiratory distress syndrome [114,115]. The preservation of SM function in old age is integral since contractile activity mobilizes leukocytes with high cytotoxicity and migratory potential (NK cells and CD8+ T lymphocytes), thus providing exchange between tissues and the circulatory system [116]. Over time, this process improves immunosurveillance and attenuates immunosenescence by preserving leukocyte function (T-cell, neutrophil, and NK cells), which lowers the immune response to bacterial challenge, enhances vaccination responses, and dampens systemic inflammation (inflammaging) [113,116,117,118,119,120]. Currently, there is no scientific data regarding the specific benefits of physical activity on the risk of developing severe COVID-19 symptoms [112]; however, there is evidence that moderate intensity PA enhances protection against other viral infections such as influenza, rhinovirus, and herpes viruses [121,122]. Home-based options are particularly appealing considering the necessity of social distancing during pandemics.

In addition to the protective effects of low-to-moderate intensity exercise, the provision of oral nutrient supplements may restore sufficient nutrient intake in older adults and malnourished patients and enhance virus protection. Inadequate nutrient intake, for example protein, calcium, vitamin D, and n-3 PUFAs, is common among older adults (as exemplified by the current RCT) [123], and it appears that malnutrition and vitamin D insufficiency are associated with worse outcomes and higher mortality rates in COVID-19 patients [72]. According to the European Society for Clinical Nutrition and Metabolism (ESPEN), preserving nutritional status and preventing or treating malnutrition may reduce complications and negative outcomes in COVID-19 patients (and presumably for other infections such as influenza) [72]. Older adults are often deficient in vitamin D, calcium, and n-3 PUFAs [123], and consume less protein than expert recommendations [56,57,58], all of which are deemed integral nutrients for muscle maintenance, viral infection prevention and nutritional management during COVID-19 [72,124,125]. Although there is limited scientific evidence to date, we predict that individuals in the higher quartiles of fitness, and with sufficient but not excess nutrient intake (neither undernutrition nor overnutrition), are more resistant to contracting SARS-CoV-2, recover faster with fewer complications, and/or respond better to vaccinations and alternative therapies.

A general limitation of our study was that we did not include women in an attempt to reduce biological variability in our data since the inclusion criteria was purposefully broad within the male gender. Secondly, although withdrawal and dropout rates were consistent with other unsupervised RE trials with limited subject interactions [36], these rates may be improved by regular visits and/or telephone counselling with participants in future RCTs [34].

## 5. Conclusions

Herein, we have demonstrated that once-daily multi-ingredient supplementation with whey, micellar casein, creatine, vitamin D3, EPA/DHA (‘Muscle5’; M5), and home-based, low-intensity resistance exercise (3 d/week), improve total lean mass, muscle fiber size, muscle-to-fat ratios, strength, performance, and overall muscle quality in free-living, physically inactive, older males. Previous RCTs have shown that an almost identical blend of nutrients augments muscle gains independently and in conjunction with supervised resistance exercise/HIIT [53], and may confer anti-inflammatory and cognitive benefits in healthy older males [69,70]. Our results also suggest that the M5 supplement may potentiate muscle gains in resistance-trained sarcopenic men, although a larger cohort, including both male and female participants, will be necessary to confirm its clinical relevance. Future studies on muscle, cognitive, and immune benefits in high-risk populations using the current low-dosage M5 iteration are warranted, particularly in women. We conclude that protein-based, multi-ingredient supplementation, such as Muscle5, is safe, well-tolerated, and an effective complement to strength training for maintenance of skeletal muscle in old age.

## Figures and Tables

**Table 1:** Anthropometry, vitals, descriptives, and co-primary outcomes at baseline and following 12 weeks of multi-component therapy (HBRE/M5) in old males.

	Old Placebo + HBRE (O-P; *n* = 16)		Old Muscle5 + HBRE (O-M5; *n* = 16)	
	Pre	Post	Pre	Post
Anthropometry, Vitals and Descriptive Data				
Age (years)	74.4 ± 1.3		77.4 ± 2.8	
Height (cm)	173.6 ± 1.6		173.1 ± 2.1	
Body Weight (kg)	82.9 ± 3.3	84.1 ± 3.5 §	87.9 ± 3.7	88.9 ± 3.8
BMI (kg/m^2^)	27.4 ± 0.9	27.8 ± 0.9 *	29.3 ± 1.1	29.6 ± 1.1
Waist-To-Hip Ratio	1.01 ± 0.02	1.00 ± 0.02	1.02 ± 0.01	1.01 ± 0.01
Resting Heart Rate (bpm) (n.p.)	68.4 ± 3.3	73.6 ± 3.0	69.2 ± 3.1	69.7 ± 4.0
Systolic Blood Pressure (mmHg)	131.8 ± 4.7	134.8 ± 3.2	135.2 ± 4.1	139.5 ± 5.6
Diastolic Blood Pressure (mmHg)	78.1 ± 2.3	76.2 ± 1.7	74.5 ± 2.0	77.7 ± 2.6
HbA1c (%)	5.8 ± 0.2	5.9 ± 0.2	6.0 ± 0.2	6.0 ± 0.2
Comorbidities	2.4 ± 0.4		2.8 ± 0.5	
Medications	3.0 ± 0.6		4.7 ± 0.9	
**DEXA**				
Muscle Mass				
TLM (kg)	53.56 ± 1.60	53.87 ± 1.54	55.80 ± 1.66	56.89 ± 1.54 *
ASM (kg)	23.66 ± 0.73	23.90 ± 0.67	24.39 ± 0.79	25.08 ± 0.81 *
ASM/h^2^ (kg/m^2^)	7.84 ± 0.21	7.93 ± 0.18	8.13 ± 0.24	8.36 ± 0.26 *
ASM/BMI (kg/[kg/m^2^])	0.87 ± 0.02	0.87 ± 0.03	0.84 ± 0.03	0.86 ± 0.03
Bone Mass				
Bone Mineral Density (g/cm^2^)	1.22 ± 0.02	1.23 ± 0.02	1.21 ± 0.02	1.20 ± 0.02
Fat Mass				
Body Fat Mass (kg)	25.81 ± 2.01	26.56 ± 2.18	28.82 ± 2.78	28.43 ± 2.84
Body Fat (%)	31.86 ± 1.40	32.31 ± 1.51	32.98 ± 2.32	32.09 ± 2.26 *
Muscle: Body Fat Ratios				
TLM/Body Fat Mass	2.24 ± 0.16	2.21 ± 0.16	2.47 ± 0.46	2.54 ± 0.45
ASM/Body Fat Mass	0.99 ± 0.08	0.99 ± 0.08	1.07 ± 0.20	1.12 ± 0.21 *
TLM/% Body Fat	1.73 ± 0.08	1.71 ± 0.08	1.92 ± 0.24	2.02 ± 0.26 *
ASM/% Body Fat	0.76 ± 0.04	0.76 ± 0.04	0.84 ± 0.11	0.89 ± 0.12 *
**Strength**				
Leg Press 1RM (kg)	145 ± 10	147 ± 11	122 ± 7	140 ± 7 *
Maximal Hand Grip (kg)	42.2 ± 2.1	44.5 ± 2.5	38.4 ± 1.6	41.4 ± 1.6 *
Isometric Knee Extension (Nm)	183.6 ± 11.5	191.5 ± 12.7	170.7 ± 10.0	179.7 ± 9.4
**Performance**				
Timed Up and Go (TUG) (s)	7.23 ± 0.29	7.16 ± 0.41	8.28 ± 0.46	7.95 ± 0.35
4-Metre Walk Test (m/s)	0.98 ± 0.05	1.04 ± 0.06	0.92 ± 0.06	0.95 ± 0.06
5-Times Sit to Stand (s)	11.68 ± 0.87	10.58 ± 0.73 *	12.41 ± 0.49	11.38 ± 0.74 *
4-Step Stair Climb (s)	2.73 ± 0.13	2.72 ± 0.13	3.16 ± 0.17	2.92 ± 0.11
SPPB Score	11.0 ± 0.4	11.3 ± 0.4	10.3 ± 0.3	10.6 ± 0.4
**Muscle Quality**				
MQ Index	48.8 ± 2.2	51.3 ± 2.7 ^§^	44.4 ± 1.6	47.7 ± 1.6 *

Abbreviations: M5; ‘Muscle5’ Multi-Ingredient Supplement, HBRE; Home-Based Resistance Exercise, TLM; Total Lean Mass, ASM; Appendicular Lean Mass, SPPB; Short Physical Performance Battery, MQ; Muscle Quality. * Significantly different pre vs. post within Treatment (*p* ≤ 0.05). ^§^ Borderline different pre vs. post within Treatment (0.05 < *p* < 0.1). All values are means ± SE.

**Table 2:** Ingredients and energy content per serving in ‘Muscle5’ and Placebo.

	Total Active Ingredient (per Serving)	Calories (kcal)
**Multi-Ingredient Supplement—‘Muscle5’**		
**Ingredients**		
^1^ Whey protein	24 g	96
^2^ Micellar casein (Calcium concentration)	16 g (416 mg)	64 (0)
^3^ Creatine	3 g	0
^4^ Vitamin D3/AM	1000 IU	0
^5^ Fish oil	10 mL	81
Eicosapentaenoic acid (EPA)	1.51 g	
Docosahexaenoic acid (DHA)	0.95 g	
Vitamin E (d-alpha tocopherol)	0.08 g	
Flavour blend	0.33 g	
**Flavoring**		
Sucrose	6.4 g	25
Stevia	100 mg	0
Chocolate flavor	2.8 g	6
**Total**		**272 kcal**
**Placebo**		
**Ingredients**		
Collagen protein	40 g	160
Sunflower oil	10 mL	81
Vitamin E (d-alpha tocopherol)	0.08 g	
Flavour blend	0.34 g	
**Flavoring**		
Sucrose	6.4 g	25
Stevia	100 mg	0
Chocolate flavor	2.8 g	6
Xanthan gum	150 mg	0
**Total**		**272 kcal**

^1^ Whey (WPI 895-I) and ^2^ micellar casein (MPI 4900) protein isolates from Fonterra. Calcium concentration: 2200 mg/100 g MPI. ^3^ creatine, ^4^ vitamin D3, and ^5^ fish oil from Creapure, Caldic, and Neptune, respectively.

**Table 3:** Macronutrient intake, physical activity levels, and compliance.

	Old Placebo + HBRE (O-P; *n* = 16)		Old Muscle5 + HBRE (O-M5; *n* = 16)	
	*Pre*	*Post*	*Pre*	*Post*
**Nutrition**				
Calories (kcal)	1852 ± 107	2239 ± 90 *	2025 ± 131	1988 ± 109
Protein (g)	81.2 ± 5.7	127.6 ± 5.2 *	88.7 ± 7.0	115.7 ± 4.5 *
Protein (g/kg body weight)	0.97 ± 0.07	1.51 ± 0.08 *	1.07 ± 0.12	1.34 ± 0.08 *
Fat (g)	77.7 ± 5.9	88.3 ± 6.5	79.6 ± 6.7	76.8 ± 8.8
Carb (g)	188.9 ± 15.0	213.7 ± 12.7	223.4 ± 20.8	199.8 ± 18.5
Vitamin D3 (mcg)	11.3 ± 5.3	5.8 ± 1.0	7.3 ± 2.3	31.3 ± 2.0 *
Calcium (mg)	782.9 ± 77.7	816.7 ± 65.1	887.1 ± 116.5	1214.4 ± 102.7 *
n-3 PUFA (g)	0.181 ± 0.058	0.224 ± 0.106	0.104 ± 0.028	2.768 ± 0.198 *
**Physical activity**				
Daily activity (steps/day)	5899 ± 772	5230 ± 483	5835 ± 808	5659 ± 592
**Compliance**				
Supplement (%)	n.a.	95.4 ± 1.9	n.a.	89.3 ± 5.0
Resistance exercise (%)	n.a.	89.1 ± 8.1	n.a.	84.1 ± 6.1

Abbreviations: M5; ‘Muscle5’ Multi-Ingredient Supplement, Carb; Carbohydrates, n-3 PUFA; omega 3 fatty acids. * Significantly different pre vs. post within Treatment (*p* ≤ 0.05). Post values account for nutritional content of the supplement (e.g., 272 kcal) and are adjusted for compliance. Not applicable (n.a.). All values are means ± SE.

**Table 4:** Muscle fiber cross-sectional areas and fiber type distributions at baseline and following 12 weeks of multi-component therapy (HBRE/M5) in old males.

	Old Placebo + HBRE (O-P; *n* = 12)			Old Muscle5 + HBRE (O-M5; *n* = 16)		
	*Pre*	*Post*	Δ (%)	*Pre*	*Post*	Δ (%)
**Cross-Sectional Areas**						
Type I (μm^2^)	5264 ± 298	6166 ± 558	19.4 ± 12.3	5163 ± 305	5714 ± 537	15.8 ± 13.2
Type IIa (μm^2^)	5052 ± 276	5817 ± 566	15.4 ± 9.8	4562 ± 236	5931 ± 579 *	30.9 ± 11.8
Type IIx (μm^2^)	4240 ± 311	4966 ± 658	17.4 ± 14.0	4409 ± 342	5497 ± 477 *	28.5 ± 9.9
**Fiber Type Distribution**						
Type I (%)	43.0 ± 3.8	41.2 ± 4.5	-	46.3 ± 4.9	38.8 ± 2.6	-
Type IIa (%)	39.6 ± 3.8	42.6 ± 2.3	-	36.3 ± 5.0	35.1 ± 3.8	-
Type IIx (%)	17.3 ± 2.9	16.1 ± 3.5	-	17.4 ± 3.4	26.2 ± 3.6 *	-

Abbreviations: M5; ‘Muscle5’ Multi-Ingredient Supplement, HBRE; Home-Based Resistance Exercise, CSAs; muscle fiber cross-sectional areas. * Significantly different pre vs. post within Treatment (*p* ≤ 0.05). All values are means ± SE.

**Table 5:** Blood markers of kidney and liver function and lipid profiles at baseline and following 12 weeks of multi-component therapy (HBRE/M5) in old males.

	Old Placebo + HBRE (O-P; *n* = 16)		Old Muscle5 + HBRE (O-M5; *n* = 16)	
	*Pre*	*Post*	*Pre*	*Post*
**Kidney Function**				
Creatinine (μmol/L)	95.7 ± 4.0	95.6 ± 4.3	91.1 ± 4.2	100.5 ± 5.9 *
**Liver Function**				
Bilirubin (μmol/L)	14.2 ± 1.6	11.9 ± 1.5§	14.8 ± 2.0	13.3 ± 1.4
Alanine Transaminase (U/L)	22.4 ± 3.0	20.9 ± 2.2	20.3 ± 3.2	20.9 ± 2.3
γ-glutamyltransferase (U/L)	28.0 ± 4.3	26.3 ± 3.1	32.3 ± 5.3	31.0 ± 4.8
**Inflammation**				
C-reactive Protein (mg/L)	4.0 ± 2.0	3.5 ± 1.4	2.0 ± 0.5	2.7 ± 0.8
**Lipid Profile**				
Cholesterol (mmol/L)	3.9 ± 0.2	3.9 ± 0.2	4.4 ± 0.3	4.2 ± 0.3
Triglycerides (mmol/L)	1.1 ± 0.1	1.2 ± 0.1	1.5 ± 0.2	1.4 ± 0.2
HDL Cholesterol (mmol/L)	1.2 ± 0.1	1.2 ± 0.1	1.1 ± 0.1	1.1 ± 0.1
LDL Cholesterol (mmol/L)	2.1 ± 0.2	2.2 ± 0.2	2.6 ± 0.3	2.4 ± 0.3
Non-HDL Cholesterol (mmol/L)	2.7 ± 0.2	2.7 ± 0.2	3.3 ± 0.3	3.1 ± 0.3
Total Cholesterol:HDL Ratio	3.2 ± 0.2	3.3 ± 0.2	4.1 ± 0.3 ^§^	3.9 ± 0.3

Abbreviations: M5; ‘Muscle5’ Multi-Ingredient Supplement, HBRE; Home-Based Resistance Exercise. * Significantly different pre vs. post within Treatment (*p* ≤ 0.05). ^§^ Significantly different from Placebo and Young Controls. All values are means ± SE.

## Supplementary Materials

The following are available online at https://www.mdpi.com/2072-6643/12/8/2391/s1, Figure S1: Immunofluorescence detection of myosin heavy chains (representative images), Table S1: Anthropometry, vitals, descriptives, and co-primary outcomes at baseline and following 12 weeks of multi-component therapy (HBRE/M5) in sarcopenic males.
