# Supplementary material for: A Five-Ingredient Nutritional Supplement and Home-Based Resistance Exercise Improve Lean Mass and Strength in Free-Living Elderly
Source: Nutrients. 2020 Aug 10;12(8):2391. doi: 10.3390/nu12082391 (PMC7468764; doi:10.3390/nu12082391)
Supplement: Supplementary file 1 [file nutrients-12-02391-s001.zip › TABLE S1. SARCOPENIA SUBJECTS.docx]

|  | **Sarcopenic All**  **(*n* = 7)** | | **Sarcopenic Placebo + HBRE**  **(*n* = 3)** | | **Sarcopenic M5 + HBRE**  **(*n* = 4)** | |
| --- | --- | --- | --- | --- | --- | --- |
|  | *Pre* | *Post* | *Pre* | *Post* | *Pre* | *Post* |
| **Anthropometry, Vitals and Descriptive Data** |  |  |  |  |  |  |
| Age (years) | 77.3 ± 3.2 | - | 75.7 ± 4.7 | - | 78.5 ± 5.0 | - |
| Height (cm) | 174 ± 2.2 | - | 175 ± 5.4 | - | 173 ± 1.4 | - |
| Body Weight (kg) | 81.0 ± 5.5 | 82.6 ± 6.2§ | 77.0 ± 12.4 | 79.4 ± 14.1 | 84.1 ± 3.2 | 85.1 ± 3.6 |
| **DEXA** |  |  |  |  |  |  |
| *Muscle Mass* |  |  |  |  |  |  |
| TLM (kg) | 49.09 ± 2.86 | **50.72 ± 3.12*** | 49.57 ± 6.61 | 51.20 ± 7.50 | 48.72 ± 1.86 | **50.37 ± 1.19*** |
| ASM (kg) | 20.65 ± 1.00 | **21.54 ± 1.00*** | 20.19 ± 2.41 | **21.14 ± 2.33*** | 20.99 ± 0.49 | **21.86 ± 0.54*** |
| ASM/h^2^ (kg/m^2^) | 6.80 ± 0.21 | **7.08 ± 0.18*** | 6.51 ± 0.42 | **6.84 ± 0.37*** | 7.03 ± 0.05 | **7.27 ± 0.06*** |
| *Fat Mass* |  |  |  |  |  |  |
| Body Fat (%) | 35.81 ± 2.83 | 35.03 ± 2.48 | 31.93 ± 2.87 | 31.87 ± 2.49 | 38.73 ± 4.21 | **37.40 ± 3.83*** |
| *Muscle : Body Fat Ratios* |  |  |  |  |  |  |
| TLM/% Body Fat | 1.41 ± 0.12 | **1.48 ± 0.11*** | 1..55 ± 0.14 | 1.59 ± 0.12 | 1.31 ± 0.19 | **1.39 ± 0.19*** |
| ASM/% Body Fat | 0.59 ± 0.05 | **0.63 ± 0.04*** | 0.64 ± 0.07 | 0.66 ± 0.05 | 0.56 ± 0.07 | **0.60 ± 0.07*** |
| **Fiber Cross-Sectional Areas** |  |  |  |  |  |  |
| Type I (µm^2^) | 5329 ± 595 | 5728 ± 556 | 5019 ± 154 | 4972 ± 752 | 5639 ± 1285 | 6483 ± 641 |
| Type IIa (µm^2^) | 4553 ± 592 | **5703 ± 960*** | 4369 ± 1006 | 4831 ± 1295 | 4737 ± 839 | 6574 ± 1473§ |
| Type IIx (µm^2^) | 3523 ± 331 | 4264 ± 410§ | 3125 ± 381 | 3770 ± 563 | 3921 ± 548 | **4758 ± 528*** |
| **Strength** |  |  |  |  |  |  |
| Leg Press 1RM (kg) | 100 ± 11 | 113 ± 12§ | 104 ± 19 | 104 ± 19 | 97 ± 17 | 122 ± 16§ |
| Maximal Hand Grip (kg) | 33.8 ± 2.8 | 35.5 ± 3.7§ | 33.2 ± 4.1 | 33.6 ± 6.1 | 34.3 ± 4.6 | 37.5 ± 5.2§ |
| Isometric Knee Extension (Nm) | 135.8 ± 16.3 | 157.7 ± 14.3§ | 142.9 ± 24.1 | 149.2 ± 29.2 | 130.5 ± 25.4 | 166.1 ± 9.8 |
| **Performance** |  |  |  |  |  |  |
| Timed Up and Go (TUG) (s) | 9.06 ± 0.66 | 8.67 ± 0.62 | 8.51 ± 0.76 | 8.08 ± 1.24 | 9.48 ± 1.08 | 9.25 ± 0.15 |
| 4-Metre Walk Test (m/s) | 0.77 ± 0.04 | **0.93 ± 0.05*** | 0.78 ± 0.07 | 0.97 ± 0.03§ | 0.76 ± 0.06 | 0.88 ± 0.10§ |
| 6-Metre Walk Test (m/s) | 0.96 ± 0.03 | **1.04 ± 0.04*** | 0.98 ± 0.03 | 1.06 ± 0.07 | 0.95 ± 0.06 | **1.02 ± 0.05*** |
| 5-Times Sit to Stand (s) | 15.45 ± 1.15 | 13.73 ± 1.71§ | 16.02 ± 2.59 | 14.17 ± 2.50§ | 15.01 ± 0.76 | 13.29 ± 2.85 |
| 4-Step Stair Climb (s) | 3.58 ± 0.27 | **3.12 ± 0.23*** | 3.29 ± 0.45 | 3.22 ± 0.47 | 3.80 ± 0.33 | **3.03 ± 0.16*** |
| SPPB Score | 9 ± 0.5 | 9.8 ± 1.1 | 9 ± 1.2 | 9.7 ± 1.5§ | 9 ± 0.5 | 10 ± 2 |
| **Muscle Quality** |  |  |  |  |  |  |
| MQ index | 38.4 ± 2.8 | 41.8 ± 3.8§ | 39.3 ± 4.6 | 39.4 ± 6.8 | 37.6 ± 4.9 | 44.1 ± 5.7§ |

**S1 Table. Anthropometry, vitals, descriptives, and co-primary outcomes pre and post 12 weeks of multi-component therapy in sarcopenic males.**

Abbreviations: M5; ‘Muscle5’ Multi-Nutrient Supplement, HBRE; Home-Based Resistance Exercise, TLM; Total Lean Mass, ASM; Appendicular Lean Mass, SPPB; Short Physical Performance Battery, MQ; Muscle Quality. *Significantly different pre vs. post within Treatment (P ≤ 0.05). § Borderline different pre vs. post within Treatment (P > 0.05 < 0.1). All values are means ± SE.
