# Supplementary figures and images for: A Five-Ingredient Nutritional Supplement and Home-Based Resistance Exercise Improve Lean Mass and Strength in Free-Living Elderly
Source: Nutrients. 2020 Aug 10;12(8):2391. doi: 10.3390/nu12082391 (PMC7468764; doi:10.3390/nu12082391)

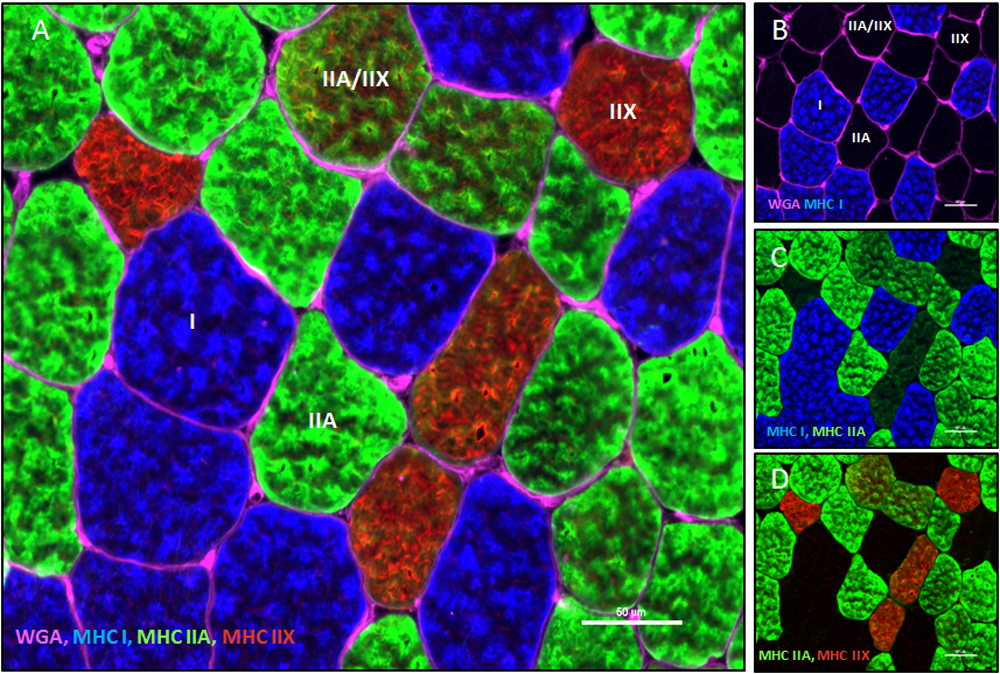

Supplement: Supplementary file 1 [file nutrients-12-02391-s001.zip › Figure S1.tif]
